# Bone destruction by receptor activator of nuclear factor κB ligand-expressing T cells in chronic gouty arthritis

**DOI:** 10.1186/ar3483

**Published:** 2011-10-13

**Authors:** Sung-Ji Lee, Kwang-Il Nam, Hye-Mi Jin, Young-Nan Cho, Song-Eun Lee, Tae-Jong Kim, Shin-Seok Lee, Seung-Jung Kee, Keun-Bae Lee, Nacksung Kim, Yong-Wook Park

**Affiliations:** 1 Department of Rheumatology, Research Institute of Medical Sciences, Brain Korea 21, Chonnam National University Medical School and Hospital, 42, Jebong-ro, Dong-gu, Gwangju 501-757, South Korea; 2 Department of Anatomy, Chonnam National University Medical School, 42, Jebong-ro, Dong-gu, Gwangju 501-757, South Korea; 3 Department of Laboratory Medicine, Chonnam National University Medical School and Hospital, 42, Jebong-ro, Dong-gu, Gwangju 501-757, South Korea; 4 Department of Orthopedic Surgery, Chonnam National University Medical School and Hospital, 42, Jebong-ro, Dong-gu, Gwangju 501-757, South Korea; 5 National Research Laboratory for Regulation of Bone Metabolism and Disease, Medical Research Center for Gene Regulation, Chonnam National University Medical School, 42, Jebong-ro, Dong-gu, Gwangju 501-757, South Korea

## Abstract

**Introduction:**

The purpose of this study was to analyze the cellular expressions of pro-resorptive cytokines in gouty tophus tissues, to determine the capacity of monosodium urate monohydrate (MSU) crystals to induce these cytokines, and to understand the mechanisms of bone destruction in chronic gout.

**Methods:**

Fourteen fixed, paraffin-embedded, uninfected tophus samples were analyzed immunohistochemically. Peripheral blood mononuclear cells (PBMCs) were cultured *in vitro *with MSU crystals, and gene expression was assessed by reverse transcription-polymerase chain reaction. *In vitro *osteoclastogenesis was performed using PBMCs and synovial fluid mononuclear cells (SFMCs).

**Results:**

CD4+ T cells, CD8+ T cells, CD20+ B cells and mast cells infiltrated tophus tissues. Tartrate-resistant acid phosphatase (TRAP)+ osteoclasts were present around tophi and in osteolytic lesions. Interleukin (IL)-1, IL-6 and tumor necrosis factor (TNF)-alpha were produced from infiltrated mononuclear cells, whereas receptor activator of nuclear factor κB ligand (RANKL) was strongly expressed in T cells. However, osteoprotegerin (OPG) was not or was weakly expressed in tophus tissues. MSU crystals induced the expressions of IL-1, IL-6, TNF-alpha and RANKL in PBMCs, but inhibited OPG expression. In addition, the pro-resorptive cytokines were highly expressed in SFMCs of gouty arthritis patients. Furthermore, *in vitro *osteoclastogenesis was enhanced in SFMC cultures, but inhibited in T cell-depleted SFMC cultures.

**Conclusions:**

Our study demonstrates that RANKL-expressing T cells and TRAP+ osteoclasts are present within gouty tophus tissues, and that infiltrating cells express pro-resorptive cytokines. Furthermore, our data show that MSU crystals have the potential to induce pro-resorptive cytokines, and T cells are involved in osteoclastogenesis in chronic gout.

## Introduction

Gout is an inflammatory arthritis caused by the deposition of monosodium urate monohydrate (MSU) crystals within joints during chronic hyperuricemia [1,2]. If left untreated, acute attacks of gout can lead to chronic gout, which is characterized by the presence of tophi, synovitis, and bone erosion [3]. Eventually, chronic tophaceous gout may lead to joint damage, deformity and disability [4].

Gouty tophus appears to be composed of chronic granulomatous lesions of mono- and multinucleated macrophages surrounding deposits of MSU crystals [5,6]. Macrophages are a major effector of inflammation, and are continuously recruited into gouty tophi [7,8]. Recently, Dalbeth *et al*. showed enhanced osteoclastogenesis in patients with chronic tophaceous gout [9], and subsequently, demonstrated that gouty tophi represent an organized chronic inflammatory tissue response involving both innate and adaptive immune cells to MSU crystals [10]. However, the cellular mechanisms by which cytokines are involved in osteoclastogenesis in response to MSU crystals remain to be clarified.

Osteoclasts are multinucleated cells, which are formed by the fusion of mononuclear progenitors of the monocyte/macrophage family [11,12], and are principally responsible for bone resorption. Osteoclasts express tartrate-resistant acid phosphatase (TRAP), and their differentiation and maturation are largely regulated by M-CSF (macrophage colony-stimulating factor), RANKL (receptor activator of nuclear factor κB ligand, also known as OPGL or TRANCE), and osteoprotegerin (OPG) [12,13]. Previous studies have demonstrated that RANKL serves as both a chemotactic and survival factor for osteoclasts [14,15], and its expression can be up-regulated by pro-resorptive cytokines, such as interleukin (IL)-1, IL-6 and tumor necrosis factor (TNF)-α [16-18].

In chronic inflammatory arthritis, such as rheumatoid arthritis (RA) and psoriatic arthritis (PsA), osteoclasts have been shown to play an important role in the pathogenesis of bone erosion [19,20]. It was demonstrated in a recent study that TRAP+ multinucleated cells (osteoclast-like cells) were present within the corona zone of gouty tophus [10]. Moreover, RANKL has been shown to mediate this osteoclastogenesis in RA and PsA [21-23], and it has been suggested that osteoclasts and the RANKL-RANK pathway are important in mediating bone erosion formation in gouty arthritis [24]. However, much less is known about the cellular origin of RANKL-RANK and the histological characteristic of TRAP+ multinucleated cells in gouty tophus. Furthermore, the role of RANKL-expressing cells during osteoclastogenesis in gouty arthritis remains to be clarified.

MSU crystals provide a proinflammatory stimulus that can initiate, amplify, and sustain an intense inflammatory response, because this stimulus induces the synthesis and release of humoral and cellular inflammatory mediators [25]. Furthermore, these crystals can interact with mast cells, endothelial cells, neutrophils, macrophages or synovial fibroblasts by phagocytosis or direct interaction with cell surface receptor, thus triggering a typical inflammatory response through the release of proinflammatory cytokines, such as IL-1β, IL-6, IL-8 and TNF-α [25-27]. These findings suggest that MSU crystals might trigger the inflammatory response observed in gouty arthritis. The aim of the present study was to analyze the cellular expressions of pro-resorptive cytokines in gouty tophus tissues, to determine the capacity of MSU crystals to induce these cytokines, and finally to understand the cellular mechanisms of bone destruction in chronic gouty arthritis.

## Materials and methods

### Sample collections

Fourteen gouty tophus tissues were obtained from eight patients with chronic gout who underwent surgery for the removal of tophi. The clinical features of the patients and tophi are summarized in Table 1. Six paired samples of peripheral blood and synovial fluid were obtained from gouty arthritis patients with knee effusion. The presence of MSU crystals in tophus and synovial fluid samples was confirmed by polarizing light microscopy. The study was approved by the Institutional Review Board of Chonnam National University Hospital, and written informed consent was obtained from all participants.

**Table 1:** Patient and tophus characteristics at the time of surgery^*a*^

Patient characteristics (*n *= 8)	
No. (%) male	7 (88)
Age, median (range) years	46 (31 to 67)
Duration of gout, median (range) years	12 (4 to 30)
No. (%) prescribed allopurinol	6 (75)
No. (%) prescribed colchicine	7 (87.5)
No. (%) prescribed nonsteroidal anti-inflammatory drugs	2 (25)
Serum urate, median (range) mg/dl	6.6 (5 to 9.8)
CRP, median (range) mg/dl	0.84 (0.2 to 4.3)
Tophus characteristics (*n *= 14)	
Duration of tophus, median (range) years	5 (1.5 to 7)
Location of tophus	
First MTP joint, No. (%)	7 (50)
Ankle joint, No. (%)	4 (29)
Elbow joint, No. (%)	2 (14)
Hand PIP joint, No. (%)	1 (7)
Indication for surgery	
Mechanical problem, No. (%)	10 (71)
Pain control, No. (%)	4 (28)
Sepsis control (infected/ulcerated tophi), No. (%)	0 (0)

CRP, C-reactive protein; MTP, metatarsophalangeal; No., number; PIP, proximal interphalangeal. ^*a*^Data are presented as medians and ranges or percentages.

### Immunohistochemistry

Gouty tophus tissue samples were fixed in neutral buffered formalin and embedded in paraffin. Bone samples were demineralized at room temperature in 10% formic acid for one week and then embedded in paraffin. Consecutive 6-μm sections were immunohistochemically stained using LSAB2 System-HRP kit (DakoCytomation, Carpentaria, CA, USA). Briefly, sections were deparaffinized, hydrated and placed in a peroxidase block solution for 10 minutes. After washing with phosphate buffered saline (PBS), sections were incubated with primary antibodies or isotype controls for 2 hours, treated with biotinylated link solution for 20 minutes, incubated in streptavidin-HRP solution for 20 minutes, treated with diaminobenzidine (DAB) chromogen solution for 10 minutes, and counterstained with hematoxylin. Sections were then mounted using Vectorshield mountant (Vector Laboratories, Burlingame, CA, USA) and analyzed by light microscopy (Olympus, Tokyo, Japan).

The following anti-human primary antibodies and dilutions were used: rabbit anti-CD3 polyclonal antibody (pAb) at a dilution of 1:100 (DakoCytomation, Produktionsvej, Denmark), mouse anti-CD4 monoclonal antibody (mAb) at 1:100 (clone IF6; Novocastra, Newcastle-upon-Tyne, UK), mouse anti-CD8 mAb at 1:100 (clone 144B; DakoCytomation), mouse anti-CD20 mAb at 1:400 (clone L26; DakoCytomation), mouse anti-CD68 mAb at 1:200 (clone Kp-1; Cell Marque, Rocklin, CA, USA), mouse anti-c-kit mAb at 1:100 (clone 104D2; DakoCytomation), mouse anti-IL-1β mAb at 1:500 (clone 8516; R&D Systems, Minneapolis, MN, USA), mouse anti-IL-6 mAb at 1:500 (clone 1936; R&D Systems), mouse anti-TNF-α mAb at 1:100 (clone 28401; R&D Systems), mouse anti-RANKL mAb at 1:100 (clone 70525, R&D Systems), mouse anti-RANK mAb at 1:400 (clone 80707; R&D Systems), and goat anti-OPG pAb at 1:20 (R&D Systems).

For TRAP staining, deparaffinized sections were hydrated, incubated in TRAP staining solution (Sigma, Poole, UK) overnight at 37°C, counterstained with hematoxylin, and then mounted using GVA mount (Zymed, San Francisco, CA, USA).

### Immunofluorescence and confocal microscopic examination

Deparaffinized sections were hydrated and then incubated with mouse anti-human RANKL mAb at 1:300 (R&D Systems) for 30 minutes at room temperature. After washing three times with PBS, sections were treated with Cy3-AffiniPure Fab fragment donkey anti-mouse IgG (Jackson Immunoresearch, West Grove, PA, USA), incubated with rabbit anti-human CD3 pAb at 1:100 (Dako) overnight at 4°C, washed with PBS, and treated with fluorescein isothiocyanate (FITC)-AffiniPure Fab fragment donkey anti-rabbit IgG (Jackson Immunoresearch). Sections were mounted using Vectorshield mountant (Vector) and analyzed by LSM 510 confocal laser microscopy (Zeiss, Jena, Germany).

### Electron microscopic examination of osteoclasts

Multinucleated giant cells were processed for morphologic evaluation by transmission electron microscopy as previously described [28]. Briefly, tissue samples were fixed in Karnovsky's fixative (2% paraformaldehyde plus 2.5% glutaraldehyde in 0.1 M sodium cacodylate buffer (pH 7.4) for two hours at 4°C. After rinsing three times for 20 minutes in the same buffer, samples were post-fixed in 1% osmium tetroxide (in 0.1 M sodium cacodylate buffer), dehydrated in a graded ethyl alcohol series, and embedded in Epon (EMbed 812; Electron Microscopy Sciences, Hatfield, PA, USA). The samples were then sectioned at 70 nm using a Leica EM UC6 ultramicrotome (Leica, Wetzlar, Germany), stained with lead citrate and alcoholic uranyl acetate, and examined under a JEM 1200EX-II transmission electron microscope (JEOL, Tokyo, Japan).

### MSU crystal synthesis

MSU crystals were prepared by recrystallization from uric acid as previously described [29]. Briefly, 0.4 gm of uric acid was dissolved in 80 ml of boiling distilled water containing 2.45 ml of 1N NaOH. After adjusting the pH of the solution to 7.2 with HCl, the solution was gradually cooled by stirring at room temperature and stored overnight at 4°C. The crystals that formed were sterilized by heating at 180°C for two hours and suspended in PBS at a concentration of 1 mg/ml. The crystals obtained by this method were of comparable size (5 to 25 μm long) and needle-shaped, negatively birefringent crystals observed by compensated polarized light microscopy. Endotoxin levels in MSU crystal preparations, as assessed by *Limulus *amoebocyte cell lysate assay (Lonza, Walkersville, MD, USA), were less than 0.01 endotoxin IU/ml.

### Isolation of peripheral blood mononuclear cells (PBMCs), synovial fluid mononuclear cells (SFMCs), monocytes and T cells

Peripheral venous blood and synovial fluid samples were collected in heparin containing tubes. PBMCs and SFMCs were isolated by density-gradient centrifugation using Ficoll-Paque Plus solution (Amersham Bioscience, Uppsala, Sweden). Monocytes and T cells were isolated from PBMCs at purities of > 95% using CD14 MicroBeads or Pan T Cell Isolation Kit II (Miltenyi Biotec, Bergisch Gladbach, Germany), respectively, and T cell depletion from SFMCs was performed using Pan T cell isolation Kit II (Miltenyi Biotec), according to manufacturers' instructions.

### Co-culture of PBMCs, monocytes and T cells with MSU crystals

Freshly isolated PBMCs, monocytes or T cells were suspended in a complete medium, which consisted of RPMI 1640, 2 mM _L_-glutamine, 100 units/ml of penicillin, and 100 μg/ml of streptomycin, supplemented with 10% fetal bovine serum (FBS; Gibco BRL, Grand Island, NY, USA), and then seeded in triplicate in six-well plates at 3 × 10^6 ^cells/well. After different concentrations of MSU crystals (0, 50, 100, and 250 μg/ml) were added directly to the culture plates, cells were cultured in completed media for the indicated times (4 hours for IL-1α, IL-1β, IL-6, and TNF-α, and 72 hours for RANKL and OPG) with at 37°C in a 5% CO_2 _humidified incubator. Cells were then harvested for RNA extraction and reverse transcription-polymerase chain reaction (RT-PCR) analysis.

Suspended cells were seeded in triplicate in six-well plates at 3 × 10^6 ^cells/well, and then cultured in complete media for the indicated times (4 hours for IL-1α, IL-1β, IL-6, and TNF-α, and 72 hours for RANKL and OPG) with different concentrations of MSU crystals (0, 50, 100, and 250 μg/ml) at 37°C in a 5% CO_2 _humidified incubator. Cells were then harvested for RNA extraction and reverse transcription-polymerase chain reaction (RT-PCR) analysis.

### *In vitro *differentiation of PBMCs, SFMCs and T cell-depleted SFMCs into osteoclasts

PBMCs, SFMCs and T cell-depleted SFMCs were differentiated to osteoclasts as described previously [9,22,30]. Briefly, freshly isolated PBMCs, SFMCs and T cell depleted SFMCs from patients with gouty arthritis were seeded in a 96-well plate at 3 × 10^5 ^cells/well, and then cultured for 10 days in α-minimum essential medium (α-MEM; HyClone Laboratories, Logan, UT, USA) supplemented with 10% FBS in the presence of a mixture of M-CSF (30 ng/ml; PeproTech, London, UK) and different concentrations (0, 10, and 100 ng/ml) of RANKL (PeproTech). The culture medium was replaced every three days. The cells were fixed and stained for TRAP (Sigma, St. Louis, MO, USA). TRAP+ cells with three or more nuclei were defined as osteoclasts, and the number of osteoclasts was counted in triplicate.

### RT-PCR and real-time PCR

RT-PCR and real-time PCR were performed as previously described [31,32]. Briefly, total RNA was extracted from cultured cells using TRIzol (Invitrogen, Carlsbad, CA, USA). First-strand cDNA was transcribed from 1 μg of RNA using Superscript RT (Invitrogen), according to the manufacturer's instructions. To determine the expression levels of specific genes and of glyceraldehyde-3-phosphate dehydrogenase (GAPDH) (used as an internal control), PCRs were performed using QuantiTect SYBR Green PCR kits (Qiagen, Valencia, CA, USA) in triplicate in a Rotor-Gene 3000 (Corbett Research, Mortlake, NSW, Australia), using the following conditions; 15 minutes at 95°C, followed by 40 amplification cycles of 95°C for 30 seconds, 58°C for 30 seconds, and 72°C for 30 seconds. All quantitations were normalized versus endogenous GAPDH. The relative quantitation value of each target gene as compared with the calibrator for that target was calculated using 2^-(Ct-Cc) ^(Ct and Cc are the mean threshold cycle differences after normalizing to GAPDH). Relative expression levels are presented using semilog plots. PCR products were subjected to electrophoresis and visualized by ethidium bromide staining. The sequences of the primers used were as follows: for GAPDH, 5'-CCATGGGGAAGGTGAAGGTCGGAG-3' (sense) and 5'-TCAGCAGAGGGGGCAGAGATGATG-3' (antisense); for IL-1α, 5'-CCAAGATGAAGACCAACCAGTGC-3' (sense) and 5'-TAGTGCCGTGAGTTTCCCAGAAG-3' (antisense); for IL-1 β, 5'-TGGCTTATTACAGTGGCAATGAGG-3' (sense) and 5'-GTAGTGGTGGTCGGAGATTCGTAG-3' (antisense); for IL-6, 5'-TGGTGTTGCCTGCTGCCTTC-3' (sense) and 5'-CCAGTGCCTCTTTGCTGCTTTC-3' (antisense); for TNF-α, 5'-GGCTCCAGGCGGTGCTTG-3' (sense) and 5'-GGGCTACAGGCTTGTCACTCG-3' (antisense); for RANKL, 5'-GCAGAGAAAGCGATGGTGGATG-3' (sense) and 5'-GGGATGTCGGTGGCATTAATAGTG-3' (antisense); and for OPG, 5'-ATGCAACACACGACAACATA-3' (sense)and 5'-GTTGCCGTTTTATCCTCTCT-3' (antisense).

### Statistical analysis

Data are shown as median (range) or mean (standard error of measurement, SEM). Wilcoxon signed rank test was used for the comparison of changes in osteoclastogenesis of SFMCs after T cell depletion. *P-*values less than 0.05 were considered statistically significant. All statistical analyses were performed using SPSS version 17.0 software (SPSS, Chicago, IL, USA).

## Results

### Infiltration of chronic inflammatory cells into gouty tophus tissues

**Figure 1:**
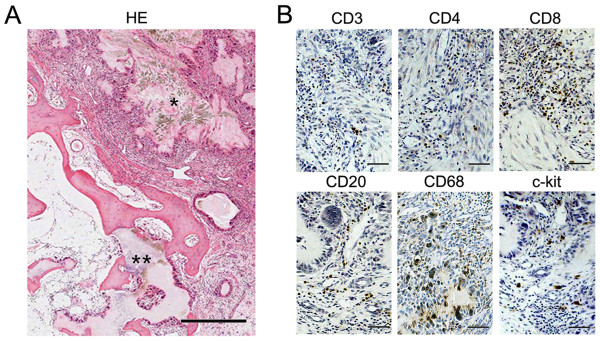
**Infiltration of chronic inflammatory cells in gouty tophus tissues**. **(a) **Tophus tissue sections from patients with chronic gouty arthritis were processed for hematoxylin-eosin (HE) staining. * indicates monosodium urate monohydrate (MSU) crystals, and ** represents osteolytic lesions. **(b) **Serial sections were processed for immunohistochemical staining using anti-CD3, anti-CD4, anti-CD8, anti-CD20, anti-CD68, and anti-c-kit antibodies (brown). Scale bar in a = 500 μm; scale bars in b = 50 μm.

Tophus samples were processed for hematoxylin-eosin (H&E) and immunohistochemical staining (Figure 1). Tophus lesions were observed within or around bone tissues. Osteolytic lesions were observed in close proximity to tophi, most of which were surrounded by mononucleated or multinucleated giant cells. Many inflammatory cells were found to have infiltrated stroma in tophi (Figure 1A), and immunohistochemistry showed that these infiltrated cells stained positively for CD3, CD4, CD8, CD20, or c-kit (a marker of mast cells). Furthermore, CD68 was strongly expressed in tophus-lining multinucleated giant cells and in some infiltrating cells (Figure 1B).

### CD68+ and TRAP+ multinucleated cells in gouty tophus tissues

**Figure 2:**
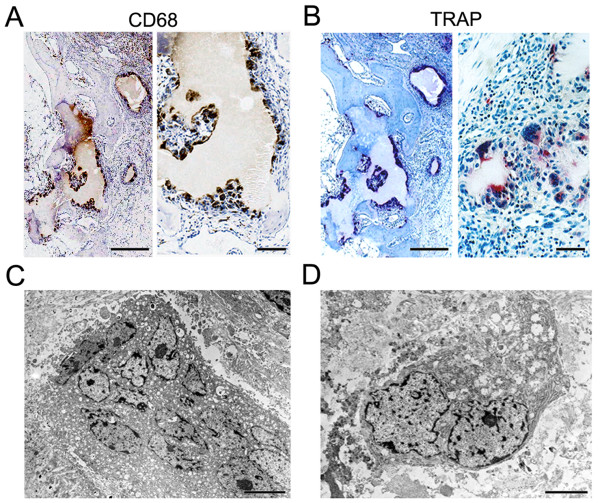
**Localization of CD68+ and tartrate-resistant acid phosphatase (TRAP)+ cells in gouty tophus tissues**. **(a) **Tophus tissue sections from patients with chronic gouty arthritis were processed for immunohistochemical staining using anti-CD68 antibody (brown). **(b) **Serial sections were stained for TRAP (red). **(c, d) **Electron microscopic findings of CD68+ cells. Scale bars in left panels of a and b = 500 μm; scale bars in right panels of a and b = 50 μm; scale bars in c and d = 5 μm.

To identify osteoclasts in tophi, TRAP staining was performed on serial tissue sections. In contrast to CD68, which was found to be expressed by immunohistochemistry in tophus-lining cells and some stromal tissue-infiltrating cells, TRAP was strongly expressed in multinucleated giant cells, which were located around tophi and in osteolytic lesions, but was not expressed in stromal tissue-infiltrating cells (Figure 2A, B). Next, we examined the ultrastructure of CD68+ and TRAP+ multinucleated giant cells around tophi by electron microscopy. These cells had variable numbers of nuclei (up to 20 nuclei), several Golgi complexes, rough endoplasmic reticulum, and numerous vesicles, which are characteristics of osteoclasts. However, no ruffled border or clear zone, characteristic of mature osteoclasts, was observed (Figure 2C, D). Collectively, these observations suggest that TRAP+ multinucleated osteoclast-like cells dominate in gouty tophi.

### Expression of pro-resorptive cytokines, RANKL and RANK in gouty tophus tissues

**Figure 3:**
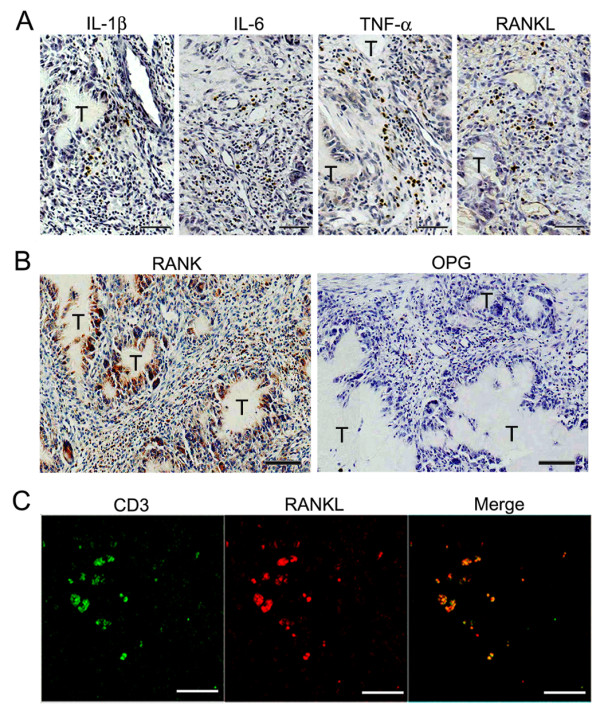
**Expression of pro-resorptive cytokines in gouty tophus tissues**. **(a, b) **Tophus tissue sections from patients with chronic gouty arthritis were processed for immunohistochemical staining using anti-interleukin (IL)-1β, anti-IL-6, anti-tumor necrosis factor (TNF)-α, anti-receptor activator of nuclear factor κB (RANK), anti-osteoprotegerin (OPG), and anti-receptor activator of nuclear factor κB ligand (RANKL) antibodies (brown). T, gouty tophus. **(c) **Immunofluorescent stains for CD3 (green) and RANKL (red) in tophaceous gout tissues. Scale bars in a, b, and c = 50 μm.

To examine the expressions of pro-resorptive cytokines in tophi, we performed immunohistochemistry using anti-IL-1β, anti-IL-6, anti-TNF-α, and anti-RANKL antibodies. These cytokines were found to be expressed in both peritophi and stromal infiltrates (Figure 3A). Next, RANK and OPG staining was performed to identify the RANKL-OPG-RANK axis within the tophus. RANK was strongly expressed in multinucleated giant cells, which were located around tophi and in osteolytic lesions. In contrast to TRAP, RANK was also expressed in some stromal tissue-infiltrating cells. However, OPG was sparsely expressed in some mononuclear cells (Figure 3B).

It has been reported that activated T cells strongly express RANKL in inflammatory regions [33,34]. Therefore, to investigate whether RANKL is expressed in T cells in tophi, tissue sections were stained by double-immunofluorescent labeling and examined by confocal microscopy. We found that RANKL-expressing cells were positive for CD3, which suggested that CD3+ T cells are cellular origin of RANKL (Figure 3C).

### Induction of pro-resorptive cytokines by MSU crystals

**Figure 4:**
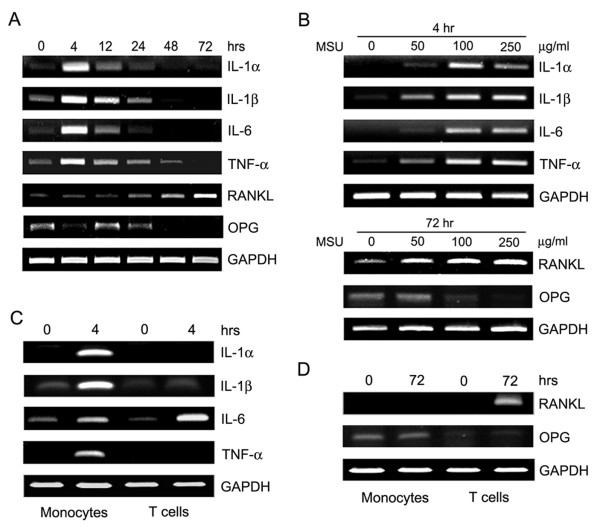
**Expression of pro-resorptive cytokines by monosodium urate monohydrate (MSU) crystals**. **(a) **Freshly isolated peripheral blood mononuclear cells (PBMCs) from healthy control subjects were cultured for the indicated times (hours) in the presence of MSU crystals (100 μg/ml). **(b) **PBMCs from same donors were cultured for the indicated times (that is, 4 hours for cytokines and 72 hours for receptor activator of nuclear factor κB ligand RANKL) at various MSU crystal concentration (μg/ml). **(c, d) **Monocytes and T cells were isolated from PBMCs by magnetic-activated cell sorting. Freshly isolated monocytes and T cells from healthy control subjects were cultured for the indicated times (hours) in the presence of MSU crystals (100 μg/ml). Total RNA was collected at each time point and at the different MSU crystal concentrations. Reverse transcription-polymerase chain reaction (RT-PCR) was performed to determine the expressions of interleukin (IL)-1α, IL-β, IL-6, tumor necrosis factor (TNF)-α, osteoprotegerin (OPG), and RANKL. Results are representative of three independent experiments.

Given our observation that pro-resorptive cytokines are expressed in gouty tophi, we investigated the ability of MSU to induce the cytokines involved in osteoclastogenesis (Figure 4). Freshly isolated PBMCs from healthy donor subjects were stimulated with MSU crystals for the indicated times, and the mRNA levels of several pro-resorptive cytokines were determined by RT-PCR. MSU crystals were found to strongly induce IL-1α, IL-1β, IL-6 and TNF-α after four hours and their expressions gradually decreased. In contrast, RANKL was induced by MSU at 24 hours and then gradually increased up to 72 hours. However, OPG was expressed at baseline and down-regulated by MSU crystals (Figure 4A). Next, we cultured PBMCs for the indicated times with increasing concentrations of MSU crystals, and examined the mRNA expressions of IL-1α, IL-1β, IL-6, TNF-α, RANKL and OPG. MSU crystals were found to induce these cytokines dose-dependently with the exception of OPG (Figure 4B). Collectively, our data suggest that MSU crystals can induce pro-resorptive cytokines and inhibit OPG expression.

To identify the cellular source of the pro-resorptive cytokines induced by MSU, monocytes and T cells isolated from healthy control subjects by magnetic-activated cell sorting were stimulated with MSU crystals for the indicated times. The mRNA levels of the pro-resorptive cytokines were then determined by RT-PCR. It was found that IL-1α, IL-1β and TNF-α were primarily produced by monocytes, but that IL-6 was produced by monocytes and T cells, and RANKL was produced by T cells alone (Figure 4C, D).

### Expression of pro-resorptive cytokines in the synovial fluid of patients with gouty arthritis

**Figure 5:**
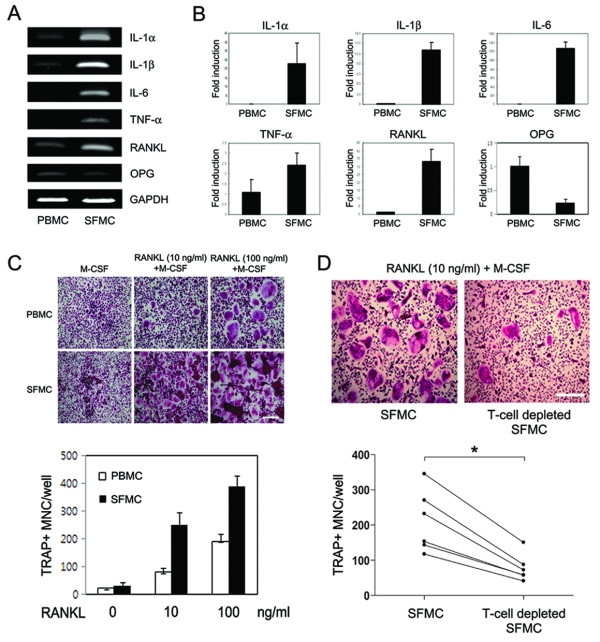
**Pro-resorptive cytokine expression and osteoclastogenesis in the synovial fluid of patients with gouty arthritis**. Six paired samples of peripheral blood and synovial fluid were obtained from gouty arthritis patients with knee effusion. **(a, b) **Expression of mRNA for interleukin (IL)-1α, IL-β, IL-6, tumor necrosis factor (TNF)-α, receptor activator of nuclear factor κB ligand (RANKL) and osteoprotegerin (OPG) in peripheral blood mononuclear cells (PBMCs) and synovial fluid mononuclear cells (SFMCs). Reverse transcription-polymerase chain reaction (RT-PCR) (a) and real-time PCR (b) were performed to determine the mRNA expression of each gene. **(c, d) **T cell involvement in osteoclastogenesis of SFMCs from patients with gouty arthritis. PBMCs, SFMCs, and T cell-depleted SFMCs (3 × 10^5 ^cells/well) were cultured for 10 days in the presence of macrophage colony-stimulating factor (M-CSF; 30 ng/ml) at the different RANKL concentrations and then were stained for tartrate-resistant acid phosphatase (TRAP). TRAP-positive multinucleated cells (MNCs) were counted in triplicate. Results are representative of six independent experiments (a and upper panels in c and d). Data in b and lower panel in c are means ± SEMs. Scale bars in c and d = 50 μm. * = *P *< 0.05, by Wilcoxon signed rank test.

To examine the expressions of IL-1α, IL-1β, IL-6, TNF-α, RANKL and OPG in patients with gouty arthritis, six paired samples of peripheral blood and synovial fluid were obtained from gouty arthritis patients with knee effusion, and mRNA levels were determined by RT-PCR and real-time PCR. MSU crystals were detected in synovial fluid samples by compensated polarized light microscopy (data not shown). As was expected, all five pro-resorptive cytokines, such as IL-1α, IL-1β, IL-6, TNF-α and RANKL, with the exception of OPG, were found to be strongly expressed in SFMCs, but to be only weakly or not expressed in PBMCs (Figure 5A, B).

### T cell-mediated osteoclastogenesis of SFMCs from patients with gouty arthritis

To examine whether increased pro-resorptive cytokines in the synovial fluid of patients with gouty arthritis have influence on osteoclast formation, osteoclasts were generated from PBMCs and SFMCs *in vitro *as described in Patients and Methods. Following culture with M-CSF only, no differences were found between the numbers of TRAP+ multinucleated cells generated from PBMCs and SFMCs. However, SFMCs were found to have the preferential ability to form osteoclast in the presence of M-CSF and RANKL as compared with PBMCs (Figure 5C).

Next, to investigate the role of synovial fluid T cells in osteoclastogenesis, we established T cell-depleted SFMC cultures from six patients with gouty arthritis. TRAP+ multinucleated cells (mean ± SEM cell number 210 ± 36 cells/well) were efficiently generated from SFMCs of gouty arthritis patients in the presence of 30 ng/ml of M-CSF and 10 ng/ml of RANKL. In contrast, the formation of TRAP+ multinucleated cells in T cell-depleted SFMC cultures was significantly reduced (mean ± SEM cell number 78 ± 16 cells/well) (*P *= 0.028) (Figure 5D). These findings suggest that synovial fluid T cells are involved in osteoclastogenesis of SFMCs from patients with gouty arthritis.

## Discussion

The present study showed that various chronic inflammatory cells infiltrated gouty tophi and that cytokines, which induce bone resorption, were produced by these cells. In addition, RANKL-expressing T cells and TRAP+ multinucleated osteoclasts (both key players in osteoclastogenesis) were observed in tophi samples. These findings suggest that gouty tophi promote bone resorption in chronic gout. Furthermore, our *in vitro *experiments showed that MSU crystals induced IL-1, IL-6, TNF-α and RANKL, and all were found to be highly expressed in the SFMCs of patients with gouty arthritis. In addition, *in vitro *osteoclastogenesis was found to be T cell-dependent. Thus, our study demonstrates that chronic inflammatory responses to MSU crystals contribute to tophus formation and bone destruction in chronic gout.

Gouty tophus has a complex, organized microstructure. Acini of macrophages were found to have formed around MSU crystals, and surrounding interstitial tissues were diffusely infiltrated by chronic inflammatory cells, including T cells, B cells, and mast cells. This microstructure of tophus has also been reported previously [5,7,10]. Interestingly, we found a predominance of CD8+ T cells over CD4+ T cells in tophi as opposed to the slight augmentation of CD4+ T cells over CD8+ T cells reported in rheumatoid synovium [35,36]. Furthermore, the arrangements of infiltrating cells in tophi were found to resemble a subtype of rheumatoid synovitis (diffuse synovitis) [37,38].

In the present study, CD68+RANK+TRAP+ osteoclasts were observed in osteolytic lesions and, interestingly, we also observed CD68+RANK+TRAP+ multinucleated cells adjacent to MSU crystals. Electron microscopic observations revealed that these cells had the cytological features of osteoclasts, that is, one or more perinuclear Golgi apparatus, vesicular or tubular membrane-bounded granules, rough endoplasmic reticulum, and many nuclei, but they did not have the ruffled border or sealing zone. The defect of sealing zone in these osteoclast-like cells might be due to the absence of signal molecules provided by bone matrix. It has been previously suggested that preosteoclasts may differentiate into osteoclasts by direct contact with bone surface exposed by osteoblast detachment [39]. Thus, our data indicate that TRAP+ osteoclast-like cells around tophi have the potential to become authentic mature osteoclasts and contribute to bony erosion in chronic gout.

Our interesting and novel observation concerned the presence of RANKL-expressing T cells in gouty tophus tissues. RANKL and OPG are key regulators of osteoclastogenesis [12], and it has been previously reported that RANKL-expressing T cells are involved in osteoclastic bone resorption in RA patients and in animal models of inflammatory arthritis [33,34]. In the present study, we demonstrated that RANKL was expressed in the tissue-infiltrated T cells. In contrast, OPG was found to be not or to be weakly expressed in the tophus tissues. In addition, proinflammatory cytokines (IL-1, IL-6, and TNF-α) were found to be expressed in infiltrating mononuclear cells. These cytokines are also known to participate in bone resorption, and to potently induce and accelerate RANKL signaling [40]. Accordingly, the expression of RANKL and proinflammatory cytokines in the tophus tissues strongly supports the histological evidence of osteoclastogenesis in chronic gout.

Our *in vitro *experiments demonstrate that MSU crystals induce the proinflammatory cytokines, IL-1, IL-6 and TNF-α, which are also expressed in tophus tissues. These cytokines were strongly induced after four hours in monocytes, indicating that MSU crystals induce innate immune responses, which is consistent with previous findings [41-43]. In addition, these cytokines are also present in the joint fluids of patients with gouty arthritis. SFMCs, which are under the milieu of various proinflammatory cytokines, showed more enhanced osteoclastogenesis than PBMCs, which findings are in agreement with data from Dalbeth *et al*. [9]. The finding that MSU crystals can induce RANKL expression in peripheral blood T cells is also interesting. In contrast to the early induction of proinflammatory cytokines (that is, IL-1, IL-6 and TNF-α), RANKL expression peaked after stimulation with MSU crystals for 72 hours, and RANKL was predominantly expressed by T cells, not monocytes. These findings are relevant to those of a recent study, which showed that uric acid can directly promote T cell activation in an antigen-independent manner [44]. To investigate the biological relevance of T cells in osteoclastogenesis of gouty arthritis, we evaluated osteoclastogenesis in T cell-depleted SFMC cultures. As shown in Figure 5C, osteoclasts were not efficiently differentiated from SFMCs in culture with M-CSF only, suggesting that endogenous RANKL in SFMCs is not enough to generate osteoclasts. Thus, 10 ng/ml of soluble RANKL was added in this experiment (Figure 5D). As compared with SFMCs cultures, osteoclastogenesis in T cell-depleted SFMC cultures was reduced by approximately 60%, not fully because of soluble RANKL (Figure 5D). These findings suggest that T cells, at least, in part play a role in osteoclast formation. Furthermore, it has been previously reported that activated T cells are involved in osteoclastogenesis through RANKL [33], and RANKL was found to be strongly expressed in SFMCs (Figure 5A, B). Thus, endogenous RANKL in synovial fluid T cells could contribute to osteoclastogenesis. Our results are consistent with literature data demonstrating that T cells are involved in the osteoclastogenesis of chronic inflammatory arthritis [30,34]. Taken together, our data suggest that the induction of pro-resorptive cytokines and formation of RANKL-expressing T cells by MSU crystals contribute to osteoclastogenesis in chronic gouty arthritis.

Our histological data show that adaptive immunity is related to the development of gouty tophi. In particular, T cells and B cells were found to have diffusely infiltrated around the tophi. Cells of the monocyte and macrophage lineages have previously been implicated in the development of bone erosion in gout [8,45]. In addition, chronic inflammatory conditions, such as RA and chronic tophaceous gout, can lead to the activation of circulating monocytes and promote their differentiation toward the osteoclast lineage [9,46]. Furthermore, recently, Li *et al*. demonstrated that osteoclasts function as antigen presenting cells and that they stimulate both CD4+ and CD8+ T cells [47]. Thus, based on our results and those of others [9,10,24,48], we propose a possible model of bone destruction in chronic gouty arthritis. MSU crystals induce the productions of proinflammatory cytokines, such as IL-1, IL-6, and TNF-α, by infiltrating monocytes and macrophages, and of RANKL by activated T cells. Subsequently, these events could induce osteoclast formation around tophi and in osteolytic lesions. Furthermore, TRAP+ osteoclasts (possibly derived from macrophages) present in the vicinity of tophus could participate in the recruitments of T and B cells. Thus, we propose that pro-resorptive cytokines, such as IL-1, IL-6, TNF-α and RANKL, contribute to osteoclastic bone resorption in patients with chronic gout.

## Conclusions

This study shows cellular and humoral evidence of bone destruction in gouty tophus tissues, and demonstrates that MSU crystals have the capacity to induce pro-resorptive cytokines. In addition, we report a novel observation on the implication of RANKL in the pathogenesis of gouty bone erosion. These findings provide important information for the exploration of therapeutic strategies to prevent bone destruction in chronic gout.

## Abbreviations

DAB: diaminobenzidine; FBS: fetal bovine serum; FITC: fluorescein isothiocyanate; GAPDH: glyceraldehyde-3-phosphate dehydrogenase; IL: interleukin; mAb: monoclonal antibody; M-CSF: macrophage colony-stimulating factor; MSU: monosodium urate monohydrate; OPG: osteoprotegerin; pAb: polyclonal antibody; PBMC: peripheral blood mononuclear cell; PBS: phosphate buffered saline; PsA: psoriatic arthritis; RA: rheumatoid arthritis; RANKL: receptor activator of nuclear factor κB ligand; RT-PCR: reverse transcription-polymerase chain reaction; SEM: standard error of measurement; SFMC: synovial fluid mononuclear cell; TNF: tumor necrosis factor; TRAP: tartrate-resistant acid phosphatase.

## Competing interests

The authors declare that they have no competing interests.

## Authors' contributions

SJL, KIN, HMJ, YNC and SEL were involved in collecting patient samples and performing the experiments. TJK and SSL participated in the interpretation of the data. SJK, KBL and NK helped to draft the manuscript and provided important intellectual content. YWP designed the experiments, performed data analysis and interpretation, and wrote the manuscript. All authors read and approved the final manuscript.
